# Application of machine learning to predict the occurrence of venous thromboembolism in patients hospitalized for coronary artery disease: a single-center retrospective study

**DOI:** 10.3389/fcvm.2025.1610938

**Published:** 2025-11-28

**Authors:** Yuan-Jiao Yang, Han-Bing Yan, Wen-Tao Liu, Zhi-Chao Yang, Xiao-Hui Wang, Chen Liu, Ya-Nan Zhang, Jun Wang, Jin-Peng Yao, Hui He

**Affiliations:** 1 Clinical Research Center, Liaoning Province Benxi Central Hospital, Benxi, Liaoning, China; 2 Department of Research and Development, Liaoning Province Benxi Clinical Bio-bank, Benxi, Liaoning, China; 3 Training Department, China Medical University Benxi Central Hospital Postgraduate Training Workstation, Benxi, Liaoning, China; 4 Department of Research and Development, Shenyang Kati Health Consulting Co. LTD, Shenyang, Liaoning, China

**Keywords:** coronary heart disease, venous thromboembolism, machine learning, prediction models, risk factors

## Abstract

**Background:**

This study aimed to construct a prediction model for the occurrence of venous thromboembolism (VTE) in patients hospitalized with coronary heart disease (CHD) using machine learning algorithms.

**Methods:**

Clinical data were from the medical records of CHD patients admitted to tertiary hospitals in eastern Liaoning Province between 2019 and 2024. Five machine learning algorithms—random forest (RF), classification and regression tree (CART), logistic regression (LR), logistic regression + least absolute shrinkage and selection operator (LR + LASSO), and extreme gradient boosting (XGBoost)—were used to construct predictive models. The area under the receiver operating characteristic curve (AUC), sensitivity, specificity, and accuracy were comparison metrics between different models.

**Results:**

A total of 3113 CHD inpatients were included in the study. In the internal validation set, XGBoost had the highest AUC (0.704), sensitivity (0.708), and accuracy (0.692), and RF had the highest specificity (0.706). In the time external validation set, LR + LASSO had the highest AUC (0.649), the highest specificity (0.683) for RF, and the highest sensitivity (0.682) and accuracy (0.656) for XGBoost. D-dimer, Age, and Neutrophil Count (NEUT) were the three most important relevant indicators.

**Conclusion:**

The prediction model based on machine learning algorithms for the occurrence of VTE in CHD inpatients has a specific diagnostic value. The prediction model constructed by LR + LASSO and XGBoost is more effective than the models constructed by other methods. The results of this study can provide research ideas for the clinical prevention and treatment of VTE events occurring in CHD inpatients.

## Introduction

Coronary heart disease (CHD) seriously affects human health and imposes a heavy disease burden on the global healthcare system (1). From 2012 to 2020, the mortality rate among CHD patients in China has risen steadily each year. In 2020, the mortality rate for urban CHD patients in China was 126.91 per 100,000 population, while the rate for rural CHD patients reached as high as 135.88 per 100,000 population. CHD has become a significant cause of death among the Chinese population (2).

Venous thromboembolism (VTE), which includes deep venous thrombosis (DVT) and pulmonary embolism (PE), is a common complication in hospitalized patients and the most common cardiovascular disease secondary to acute ST-elevated myocardial infarction (STEMI) and stroke (3). The estimated incidence of VTE in Europe and the United States is estimated to be 1–2/1,000 person-years (4). In Asia, which varies widely by age, gender, ethnicity, and medical conditions, the incidence of VTE is thought to be lower than in Europe and the United States (5). Some studies have reported an approximately 38-fold increased risk of VTE in hospitalized patients compared to ambulatory patients (6), and 25%–70% of VTE events are associated with hospitalization (7–9). Therefore, the occurrence of VTE in hospitalized patients is an important indicator of overall health (5).

There is a lack of epidemiological statistics on the occurrence of VTE events in CHD patients in China. According to the U.S. National Inpatient Sample Database, the incidence of VTE in hospitalized adult patients with STEMI in the U.S. from 2003 to 2013 was approximately 1%, of which STEMI patients who developed VTE accounted for 17.3% of the overall in-hospital mortality rate, which was significantly higher than that of those who did not develop VTE (8.9%). In addition, patients who developed VTE had higher hospitalization costs compared to those who did not develop VTE (10).

Higher morbidity, mortality, and healthcare expenditures warrant more attention to the occurrence of VTE events in CHD inpatients. Therefore, early detection and timely management of VTE in CHD inpatients is critical. Clinicians screen for VTE in hospitalized patients using the scores of Padua (11) and Caprini (12). Variables in these scoring systems mainly include active cancer, previous VTE, reduced activity, known thrombotic disease, trauma and/or surgery, age, cardiac and/or respiratory failure, acute myocardial infarction or ischemic stroke, acute infections and/or rheumatic diseases, clinical characteristics such as obesity and hormone therapy, body weight, and some serological and genetic markers (11, 13–16). However, the fact that some of these VTE risk factors used in the Padua and Caprini scores overlap with those of CHD and that some of the tests in the Padua and Caprini scores are not available in community health care facilities, as well as the variability in ethnogenetic characteristics and the social environment, make the existing scoring models not applicable to the entire population of patients with VTE (17).

Therefore, this study collected widely validated risk factors for VTE onset in clinical practice and reported risk factors for VTE in CHD patients as research variables. Concurrently, we gathered clinical characteristics and laboratory indicators of hospitalized CHD patients to construct the research dataset. Using this dataset as a platform, we employed various machine learning algorithms to develop predictive models for the occurrence of VTE in hospitalized patients with CHD. In order to improve the interpretability of the results, the machine learning algorithms in this study mainly included logistic regression (LR), logistic regression + least absolute shrinkage and selection operator (LR + LASSO), categorical regression tree (CART), random forest (RF), and extreme gradient boosting (XGBoost).

## Methods

### Data source

The data for this study were from the Benxi Central Hospital and the Benxi Clinical Biospecimen Repository in Liaoning Province. We collected data from patients hospitalized for CHD between 2019 and 2024. The Chinese Clinical Trial Register approved clinical study admission at https://www.chictr.org.cn/ with the registration number ChiCTR2400094214. The Ethics Committee of Benxi Central Hospital approved this study (20040809). The Ethics Committee of Benxi Central Hospital agreed to the application for exemption from informed consent for this study due to its retrospective nature, which did not require written informed consent. Patient records and information were anonymized and de-identified prior to analysis.

### Definition

The diagnosis of VTE (ICD-10, I80) was based on the CHEST Guideline and Expert Panel Report 2016 (18) and the 2020 American Society of Hematology guidelines (19). All CHD patients (ICD10, I25) included in this study underwent limb venous vascular ultrasound, and three clinically experienced physicians confirmed the diagnosis of VTE based on vascular ultrasound.

The specific diagnostic methods were as follows: the images were initially interpreted by the reporting physician and subsequently reviewed by a physician with a higher title or seniority. Both physicians had undergone professional standardized training to minimize information bias. A vascular disease specialist manually reviewed all cases in which the imaging data confirmed the presence of VTE.

### Inclusion and exclusion criteria

Inclusion criteria: (i) age ≥ 18 years; (ii) patients hospitalized with a diagnosis of CHD who underwent limb vascular ultrasound; (iii) data obtained between 2019 and 2024.

**Figure 1:**
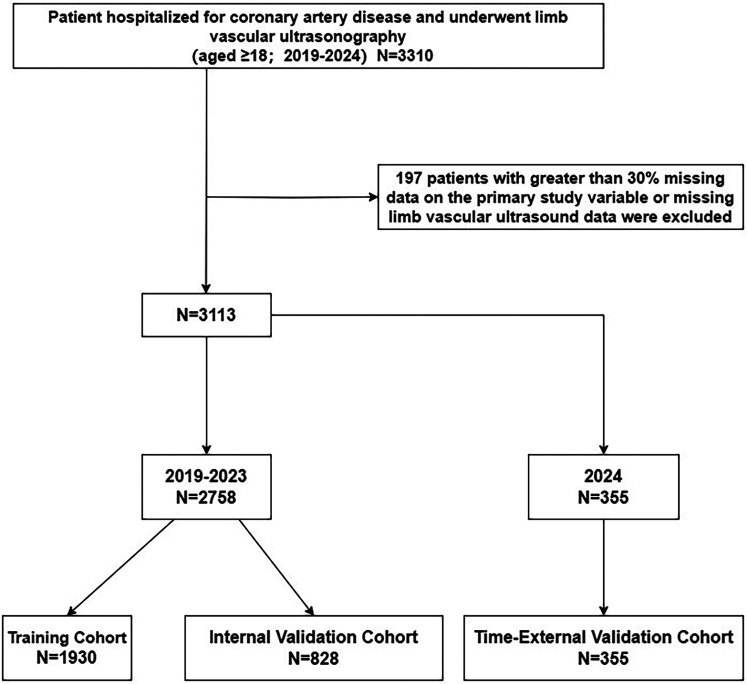
Flow of inclusions and exclusions.

Exclusion criteria: (i) patients with >30% missing data on the main study variables (20). (ii) missing limb vascular ultrasound data. The study selection process was shown in Figure 1.

### Data collection

Based on previous studies, we selected 41 risk factors that may be associated with VTE in CHD inpatients. These risk factors were from variables previously employed in published risk assessment models, such as the Caprini and Padua scores (21). We had also collected several readily available tests in primary healthcare settings (11, 13).

Forty-one risk factors included: Gender, Active Cancer, Heart Failure, Stroke, Rheumatic Disease, Myocardial Infarction, Respiratory Failure, Lung Diseases, Infections, Blood Transfusion, Trauma, Limitations of limb movement, Dyspnea, Chest Pain, Age, Diastolic Blood Pressure (DBP), Systolic Blood Pressure (SBP), Pulse Rate (PR), Body Mass Index（BMI）, Prothrombin Activity (PTA), Activated Partial Thromboplastin Time (APTT), Fibrinogen (Fib), Prothrombin Time (TT), D-dimer, Neutrophil Count (NEUT), Lymphocyte Count (LYM), Monocyte Count (MONO), Red Blood Cell Count (RBC), Hemoglobin (HGB), Red Blood Cell Distribution Width-Coefficient Of Variation (RDWCV), Red Blood Cell Distribution Width-Standard Deviation (RDWSD), Platelet Count (PLT), Uric Acid (UA), Direct Bilirubin (DBIL), Indirect Bilirubin (IBIL), Globulin (GLB), Alanine Aminotransferase (ALT), Aspartate Aminotransferase (AST), Creatinine (CRE), Blood Urea Nitrogen (BUN) And Fasting Blood Glucose (FBG).

### Statistical analyses

We used a multiple imputation method based on predicted mean matching to handle missing continuous variables (22). Predicted mean matching not only considered linear relationships between variables but also filled in missing values based on the distribution characteristics of the original data, ensuring that the distribution of the imputed data remains consistent with that of the original data. By comparing the description of the imputed data with the original data, we ensured that all imputed values were reasonable and accurate. The patients included in this study had a treatment duration spanning from 2019 to 2024, with all data sourced from the same center. There were sufficient cases for time-external validationto assess the model's stability over time (23). We divided the internal dataset (data from hospitalized patients between 2019 and 2023) and the time-external validation set (data from hospitalized patients after 2024) based on the patients' hospitalization duration (24). We split the internal dataset into a training set and an internal validation set in a 7:3 ratio (Figure 1). We used the training set to establish the predictive model. Then we validated and compared the final performance of each model in both the internal validation set and the time-external validation set. We used receiver operating characteristic (ROC) curves, area under the curve (AUC), sensitivity, specificity, and accuracy as comparison metrics to rank different models, with the AUC value being the most important ranking criterion. After selecting the optimal model, we ranked the variables in the model based on their importance to identify the key variables influencing the model, which were also important factors associated with VTE events in CHD patients hospitalized with VTE.

Continuous variables were expressed as median (interquartile range). Categorical variables were reported as percentage counts. Continuous variables were tested with the Mann–Whitney *U* test and categorical variables with the chi-square test. All statistical analyses were performed using two-sided tests, and *P* < 0.05 was considered statistically significant. Statistical analyses were performed using SPSS 26.0 and R (version 4.4.2).

## Results

### Patient characteristics

This study included a total of 3,113 hospitalized patients with CHD, among whom 474 had VTE (prevalence rate of 15.2%). The data were divided into a training set (*N* = 1,930), an internal validation set (*N* = 828), and a time-external validation set (*N* = 355). There was no statistically significant difference between the data before and after interpolation, as shown in Table 1.

**Table 1:** Comparison of continuous variables before and after interpolation.

Variables	Before interpolation	After interpolation	*P*-values
Age (IQR; years)	76.00 (67.00; 85.00)	76.00 (67.00; 85.00)	1.000
DBP (IQR; mmHg)	78.00 (70.00; 88.00)	78.00 (69.00; 88.00)	0.518
SBP (IQR; mmHg)	139.00 (124.00; 155.00)	139.00 (123.00; 155.00)	0.848
PR (IQR; bpm)	80.00 (71.00; 92.00)	80.00 (70.00; 92.00)	0.348
BMI (IQR; kg/m²)	24.20 (21.64; 26.87)	24.03 (21.48; 26.67)	0.105
PTA (IQR; %)	94.00 (84.00; 104.00)	95.00 (84.00; 105.00)	0.360
APTT (IQR; s)	36.90 (33.90; 40.50)	36.80 (33.80; 40.40)	0.409
Fib (IQR; g/L)	3.77 (3.05; 4.79)	3.74 (3.05; 4.75)	0.478
TT (IQR; s)	17.00 (16.20; 17.80)	17.00 (16.20; 17.80)	0.856
D-dimer (IQR; mg/L)	1.00 (0.46; 2.09)	0.97 (0.46; 2.02)	0.319
NEUT (IQR; × 10^9^/L)	4.95 (3.62; 7.27)	4.92 (3.60; 7.21)	0.611
LYM (IQR; × 10^9^/L)	1.36 (0.92; 1.86)	1.36 (0.93; 1.85)	0.939
MONO (IQR; × 10^9^/L)	0.48 (0.36; 0.64)	0.48 (0.36; 0.64)	0.668
RBC (IQR; × 10^12^/L)	4.16 (3.65; 4.59)	4.16 (3.66; 4.57)	0.929
HGB (IQR; g/L)	126.00 (110.00; 140.00)	126.00 (111.00; 139.00)	0.871
RDWCV (IQR; %)	13.30 (12.70; 14.10)	13.30 (12.60; 14.10)	0.581
RDWSD (IQR; fI)	44.10 (41.80; 47.30)	44.00 (41.80; 47.20)	0.704
PLT (IQR; × 10^9^/L)	201.00 (160.00; 253.00)	200.00 (160.00; 251.00)	0.684
UA (IQR; μmol/L)	346.00 (273.00; 445.00)	345.00 (272.50; 442.00)	0.858
DBIL (IQR; μmol/L)	3.00 (2.10; 4.70)	3.00 (2.10; 4.70)	0.597
IBIL (IQR; μmol/L)	6.30 (4.00; 9.10)	6.30 (4.00; 8.90)	0.733
GLB (IQR; g/L)	27.70 (25.00; 30.80)	27.60 (24.80; 30.70)	0.528
ALT (IQR; U/L)	17.00 (12.00; 26.00)	18.00 (13.00; 26.00)	0.614
AST (IQR; U/L)	21.00 (16.00; 29.00)	21.00 (16.00; 29.00)	0.877
CRE (IQR; μmol/L)	77.00 (62.00; 104.00)	77.00 (62.00; 101.00)	0.354
BUN (IQR; mmol/L)	6.93 (5.29; 9.47)	6.85 (5.29; 9.09)	0.267
FBG (IQR; mmol/L)	6.17 (5.14; 8.34)	6.18 (5.14; 8.30)	0.845

DBP, diastolic blood pressure; SBP, systolic blood pressure; PR, pulse rate; BMI, body mass index; PTA, prothrombin activity; APTT, activated partial thromboplastin time; Fib, fibrinogen; TT, thrombin time; NEUT, neutrophil count; LYM, lymphocyte count; MONO, monocyte count; RBC, red blood cell count; HGB, hemoglobin; RDWCV, red blood cell distribution width-coefficient of variation; RDWSD, red blood cell distribution width-standard deviation; PLT, platelet count; UA, uric acid; DBIL, direct bilirubin; IBIL, indirect bilirubin; GLB, globulin; ALT, alanine aminotransferase; AST, aspartate aminotransferase; CRE, creatinine; BUN, blood urea nitrogen; FBG, fasting blood glucose; IQR, interquartile range.

In the training set, there were 285 cases of VTE (prevalence of 14.8%) and 1,645 cases of non-VTE. We used PASS software to estimate the sample size based on the prevalence of the training set samples obtained. The number of samples in the training set of this study met the sample size requirements for constructing a prediction model.

The results of the training set were in Table 2. Compared with patients in the non-VTE group, patients in the VTE group had a higher proportion of females (*P* < 0.001), rheumatic diseases (*P* < 0.001), infections (*P* = 0.006), blood transfusions (*P* = 0.004), and dyspnea symptoms (*P* = 0.018).

**Table 2:** Univariate analyses of variables associated with VTE.

Variables	VTE (*N* = 285)	Non-VTE (*N* = 1,645)	*P*-values
Gender/Female (*n*, %)	160 (56.14%)	721 (43.83%)	<0.001
Active cancer (*n*, %)	7 (2.46%)	61 (3.71%)	0.290
Heart failure (*n*, %)	167 (58.60%)	911 (55.38%)	0.313
Stroke (*n*, %)	61 (21.40%)	331 (20.12%)	0.619
Rheumatic disease (*n*, %)	6 (2.11%)	25 (1.52%)	0.468
Myocardial infarction (*n*, %)	62 (21.75%)	411 (24.98%)	0.242
Respiratory failure (*n*, %)	71 (24.91%)	253 (15.38%)	<0.001
Lung diseases (*n*, %)	70 (24.56%)	381 (23.16%)	0.606
Infections (*n*, %)	161 (56.49%)	784 (47.66%)	0.006
Blood transfusion (*n*, %)	28 (9.82%)	89 (5.41%)	0.004
Trauma (*n*, %)	17 (5.96%)	100 (6.08%)	0.941
Limb limitations (*n*, %)	23 (8.07%)	109 (6.63%)	0.373
Dyspnea (*n*, %)	113 (39.65%)	534 (32.46%)	0.018
Chest pain (*n*, %)	19 (6.67%)	170 (10.33%)	0.054
Age (IQR; years)	81 (73.00, 86.00)	75 (66.00, 84.00)	<0.001
DBP (IQR; mmHg)	77 (68.00, 88.00)	78 (70.00, 87.00)	0.336
SBP (IQR; mmHg)	136 (122.50, 155.00)	140 (123.00, 155.00)	0.207
PR (IQR; bpm)	82 (72.00, 97.50)	80 (70.00, 92.00)	0.017
BMI (IQR; kg/m²)	23.44 (20.76, 26.29)	24.16 (21.56, 26.64)	0.013
PTA (IQR; %)	92 (80.00, 100.00)	96 (85.00, 106.00)	<0.001
APTT (IQR; s)	36.6 (33.60, 40.55)	36.7 (33.90, 40.40)	0.996
Fib(IQR; g/L)	3.88 (3.06, 4.86)	3.68 (3.04, 4.69)	0.317
TT (IQR; s)	17 (16.20, 17.90)	17 (16.20, 17.80)	0.940
D-dimer (IQR; mg/L)	1.93 (0.94, 4.99)	0.89 (0.43, 1.88)	<0.001
NEUT (IQR; × 10^9^/L)	5.59 (4.10, 8.48)	4.77 (3.59, 6.83)	<0.001
LYM (IQR; × 10^9^/L)	1.21 (0.82, 1.62)	1.41 (0.96, 1.92)	<0.001
MONO (IQR; × 10^9^/L)	0.48 (0.36, 0.66)	0.48 (0.36, 0.63)	0.934
RBC (IQR; × 10^12^/L)	4.02 (3.56, 4.46)	4.18 (3.70, 4.60)	<0.001
HGB (IQR; g/L)	122 (107.00, 136.00)	127 (112.00, 140.00)	<0.001
RDWCV (IQR; %)	13.5 (12.90, 14.60)	13.2 (12.60, 13.90)	<0.001
RDWSD (IQR; fI)	44.8 (42.70, 48.95)	43.8 (41.70, 47.00)	<0.001
PLT (IQR; × 10^9^/L)	200 (161.00, 255.50)	202 (160.00, 253.00)	0.940
UA (IQR; μmol/L)	340 (272.50, 445.00)	347 (273.00, 445.00)	0.795
DBIL (IQR; μmol/L)	3.3 (2.10, 5.25)	2.9 (2.10, 4.60)	0.072
IBIL (IQR; μmol/L)	6.4 (3.90, 8.80)	6.3 (4.00, 9.10)	0.778
GLB (IQR; g/L)	27.9 (25.60, 31.55)	27.4 (24.70, 30.60)	0.008
ALT (IQR; U/L)	19 (13.50, 30.00)	18 (13.00, 26.00)	0.045
AST (IQR; U/L)	22 (16.00, 29.50)	21 (16.00, 29.00)	0.647
CRE (IQR; μmol/L)	78 (61.00, 102.00)	77 (61.00, 101.50)	0.592
BUN (IQR; mmol/L)	7.46 (5.39, 10.21)	6.68(5.29, 8.99)	0.011
FBG(IQR; mmol/L)	6.4(5.33, 8.87)	6.15(5.12, 8.03)	0.023

VTE, venous thromboembolism; limb limitations, Limitations of limb movement; DBP, diastolic blood pressure; SBP, systolic blood pressure; PR, pulse rate; BMI, body mass index; PTA, prothrombin activity; APTT, activated partial thromboplastin time; Fib, fibrinogen; TT, thrombin time; NEUT, neutrophil count; LYM, lymphocyte count; MONO, monocyte count; RBC, red blood cell count; HGB, hemoglobin; RDWCV, red blood cell distribution width-coefficient of variation; RDWSD, red blood cell distribution width-standard deviation; PLT, platelet count; UA, uric acid; DBIL, direct bilirubin; IBIL, Indirect Bilirubin; GLB, Globulin; ALT, alanine aminotransferase; AST, aspartate aminotransferase; CRE, creatinine; BUN, *blood* urea nitrogen; FBG, fasting blood glucose; IQR, interquartile range.

Age (*P* < 0.001), PR (*P* = 0.017), D-dimer (*P* < 0.001), NEUT (*P* < 0.001), RDWSV (*P* < 0.001), RDWSD (*P* < 0.001), GLB (*P* = 0.008), ALT (*P* = 0.045), BUN (*P* = 0.011) and FBG (*P* = 0.023) were higher than those in the non-VTE group. The levels of BMI (*P* = 0.013), PTA (*P* < 0.001), LYM (*P* < 0.001), RBC (*P* < 0.001), and HGB (*P* < 0.001) were lower in the patients in the VTE group than those in the non-VTE group.

### Predictive effects of different models

**Figure 2:**
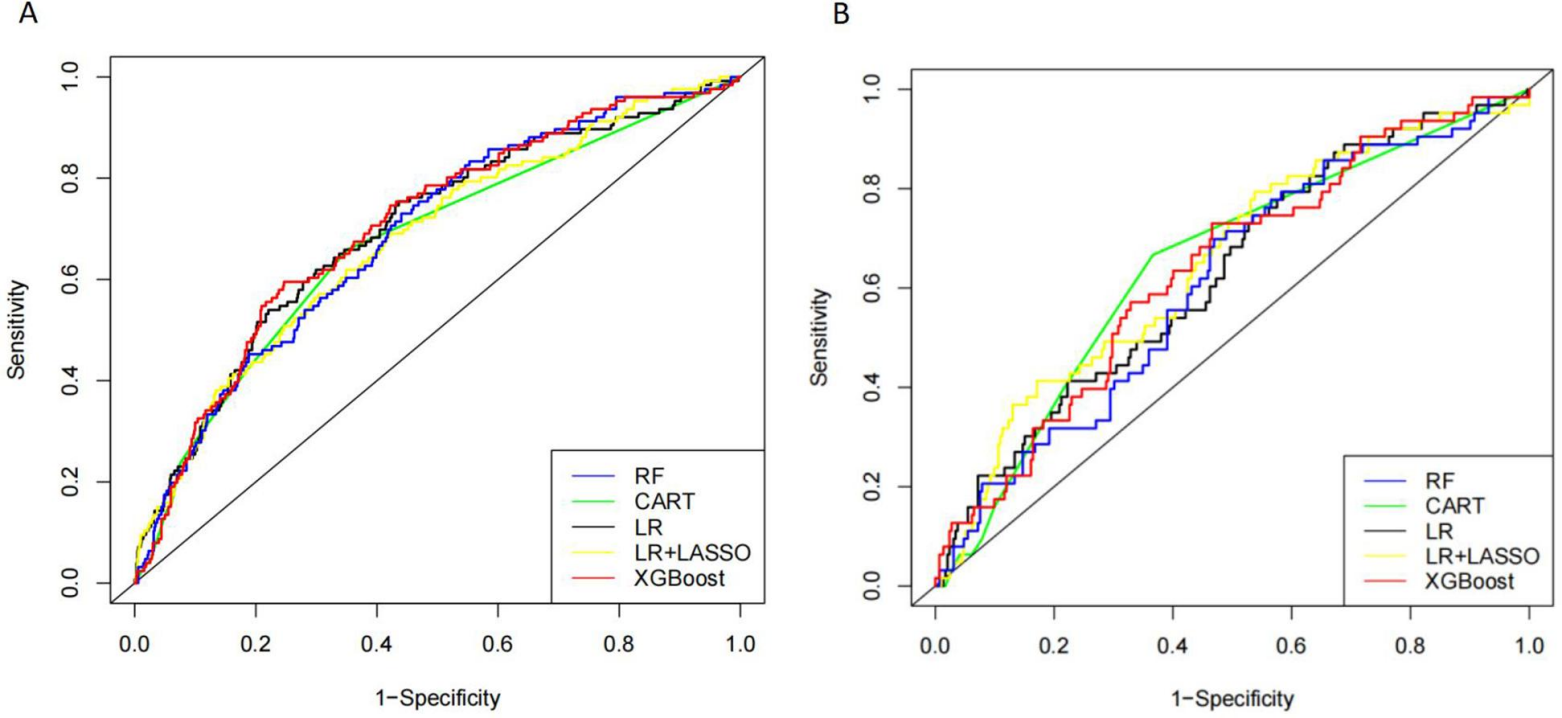
Receiver operating characteristic (ROC) curves of five different models in internal validation cohort **(A)** and time-external validation cohort **(B)**.

Five models, RF, CART, LR, LR + LASSO, and XGBoost, were used to model the prediction of VTE occurring in CHD inpatients. As shown in Figure 2, the ROC curves showed the performance results of the five different models for predicting VTE in the internal and time-external validation sets. The results showed that in the internal validation set, the XGBoost model had the best performance in predicting the occurrence of VTE events with an AUC value of 0.704. In the tine-external validation set, the LR + LASSO model performed best in predicting VTE events with an AUC value of 0.650.

As shown in Table 3, in the internal validation set, XGBoost had the highest AUC (0.704), sensitivity (0.708), and accuracy (0.692), and RF had the highest specificity (0.706). In the time external validation set, LR + LASSO had the highest AUC (0.649), the highest specificity (0.683) for RF, and the highest sensitivity (0.682) and accuracy (0.656) for XGBoost (Table 4).

**Table 3:** Detailed performance metrics for the five models in internal validation.

Model	Sensitivity	Speciﬁcity	Accuracy	AUC (95% CI)
RF	0.577	0.706	0.597	0.688 (0.639–0.737)
CART	0.65	0.659	0.651	0.671 (0.622–0.721)
LR	0.704	0.603	0.688	0.698 (0.647–0.749)
LR + LASSO	0.697	0.571	0.678	0.679 (0.627–0.730)
XGBoost	0.708	0.603	0.692	0.704 (0.655–0.754)

LR, logistic regression; CART, classification and regression tree; RF, random forest; AUC, area under the receiver operating characteristic curve; CI, confidence interval.

**Table 4:** Detailed performance metrics for the five models in external validation.

Model	Sensitivity	Speciﬁcity	Accuracy	AUC (95% CI)
RF	0.531	0.683	0.558	0.611 (0.536–0.685)
CART	0.634	0.667	0.639	0.647 (0.578–0.715)
LR	0.644	0.492	0.617	0.628 (0.553–0.702)
LR + LASSO	0.603	0.54	0.592	0.649 (0.574–0.723)
XGBoost	0.682	0.54	0.656	0.637 (0.563–0.710)

LR, logistic regression; CART, classification and regression tree; RF, random forest; AUC, area under the receiver operating characteristic curve; CI, confidence interval.

**Figure 3:**
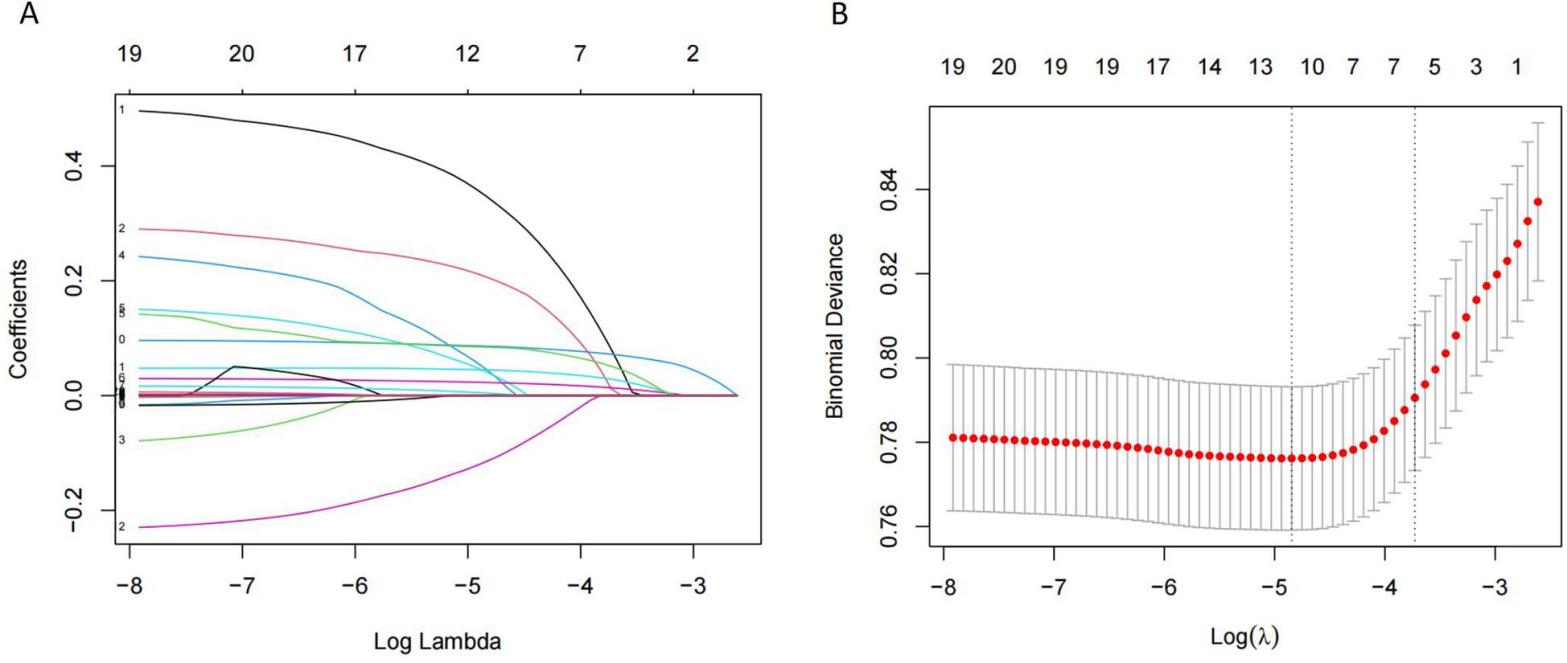
Features selection by LASSO. **(A)** LASSO coefficients profiles (*y*-axis) of the 20 features. The upper *x*-axis is the average numbers of predictors and the lower *x*-axis is the log (λ). **(B)** Ten-fold cross-validation for tuning parameter selection in the LASSO model.

**Figure 4:**
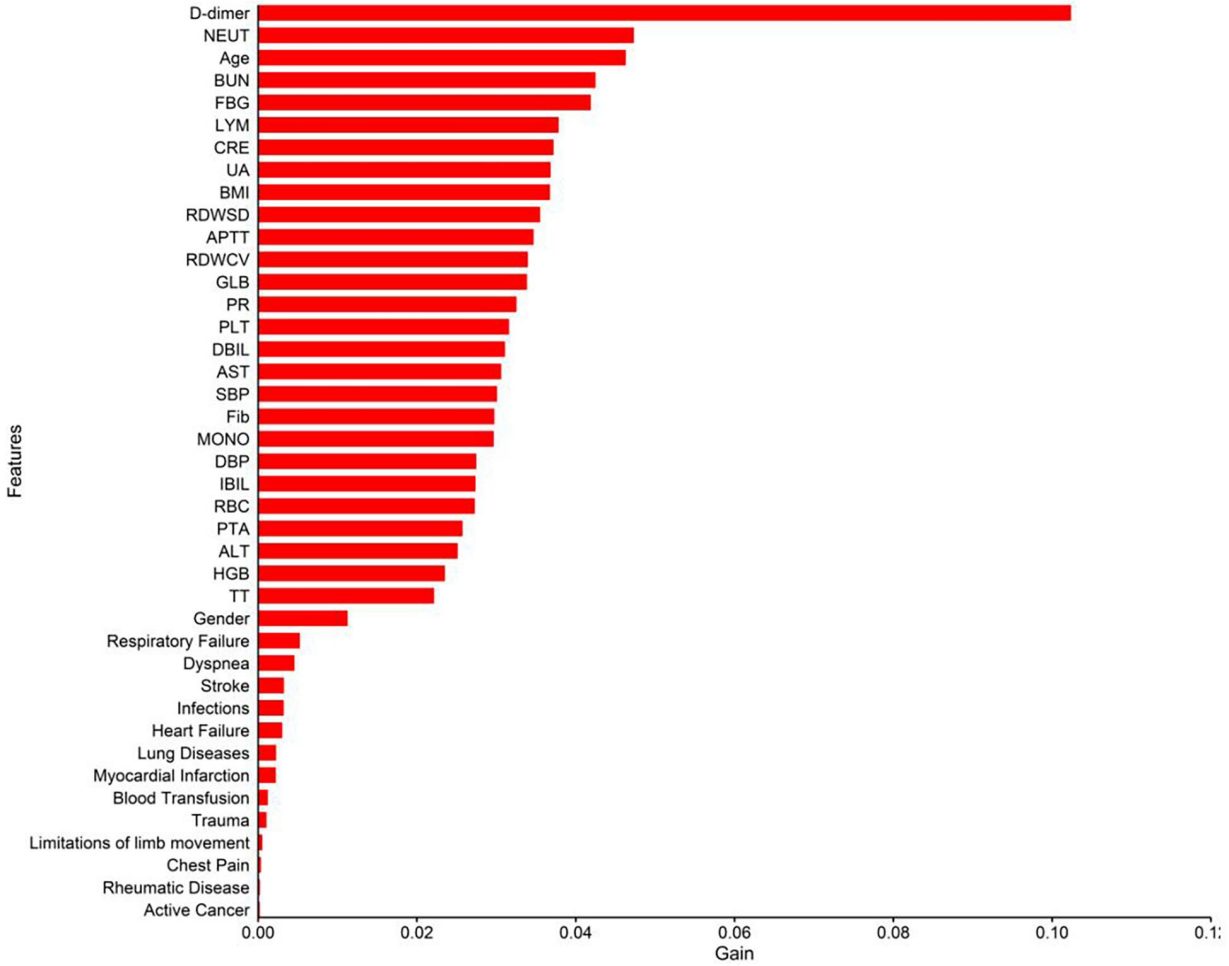
Importance analysis of indexes in XGBoost model.

Table 5 presented the order of importance of the top five feature variables in the five models in the internal and time-external validation sets. As shown in Table 5, D-dimer, NEUT, and Age were the main features in the prediction model for the occurrence of VTE in CHD inpatients. Figure 3 showed the feature screening process for the LR + LASSO model, and Figure 4 showed the feature importance ranking for the XGBoost model.

**Table 5:** Ranks of feature importance in RF, XGBoost, LR, CART, and LR + LASSO for predicting VTE.

Rank	XGBoost	LR + LASSO	LR	CART	RF
1	D-dimer	D-dimer	D-dimer	D-dimer	D-dimer
2	NEUT	Age	Age	GLB	NEUT
3	Age	NEUT	NEUT	Gender	Age
4	BUN	Gender	Gender	NEUT	BMI
5	FBG	RDWCV	MONO	Fib	BUN

LR, logistic regression; RF, random forest; CART, classification and regression tree; VTE, venous thromboembolism; BMI, body mass index; Fib, fibrinogen; NEUT, neutrophil count; MONO, monocyte count; RDWCV, red blood cell distribution width-coefficient of variation; GLB, globulin; BUN, blood urea nitrogen; FBG, fasting blood glucose.

## Discussion

A total of 3,113 CHD inpatients were included in this study, and 41 possible factors associated with the occurrence of VTE were included. Five machine learning algorithms were used to construct predictive models, and the final performance of each model was validated and compared in an internal validation set and an time-external validation cohort. The results show that the prediction models constructed by XGBoost and LR + LASSO were more effective than those constructed by the other algorithms (Tables 3, 4) (Figure 2). D-dimer, NEUT, and Age were the most important correlates of VTE in CHD inpatients (Table 5) (Figure 4).

Machine learning algorithms are a powerful data analysis tool to help in clinical decision-making and disease prevention (25–27). RF is one of the most popular machine-learning techniques for prediction problems (28). RF belongs to the category of supervised machine learning and is widely used for classification and regression. It is an easy-to-use machine-learning algorithm that produces good results without hyperparameter tuning (29). CART is a binary recursive partitioning process capable of processing continuous and nominal attributes as objectives and predictions (30). It does not need to specify the association between the independent variables and the results. The constructed tree is automatically modified to reduce the effect of noise, and the validity of the nodes is determined for the final decision (31). Although both RF and CART are decision tree algorithms, they are prone to overfitting problems due to the depth and complexity of the tree, i.e., the model may over-capture specific rules of the training data, leading to a loss of generalization (32). LR is a commonly used machine learning model in medical research for assessing associations between one or more independent variables and a binary dependent variable (33). The disadvantages of this algorithm are that it requires a large amount of training data, and the interpretation of the results is more complex (34). LASSO is a prevalent machine learning model that performs variable selection and regularisation to improve the resulting model's prediction accuracy and interpretability (35). LR + LASSO incorporates regularisation on top of linear regression, and LASSO performs variable selection and model fitting (Figure 3). XGBoost is an excellent integrated learning algorithm (36). It improves the accuracy of the output by serial integration of decision tree models and has a strong learning ability. The complexity of the model is controlled by regularisation, which helps to prevent overfitting. The essence of XGBoost is to integrate multiple weak classifiers into a single strong classifier to improve prediction accuracy (37).

Previous VTE risk prediction models have also employed the five machine learning methods examined in this study. Among multiple models predicting VTE occurrence in intensive care units, RF demonstrated the best performance (38). In pediatric oncology studies, CART models successfully predicted VTE based on risk factors (39). In a prospective cohort study, researchers developed multiple VTE risk prediction models for patients with traumatic brain injury and found that LR performed better than other models (40). In a prognostic study of VTE in allogeneic transplant patients, LASSO was the optimal machine learning model (41). During the development and validation of machine learning models for predicting VTE in hospitalized cancer patients, the XGBoost model demonstrated superior performance compared with other machine learning approaches (42).

In this study, XGBoost achieved the best results in the internal validation set, and LR + LASSO achieved the best results in the time-external validation set. Based on the order of importance of the first five characteristic variables of the first two models in the validation set, we identified D-dimer, NEUT, and age as the most important correlates of the occurrence of VTE in CHD inpatients (Table 5, Figure 4).

In patients in the VTE group, the median D-dimer was 1.93 mg/L. In contrast, in patients in the non-VTE group, the median D-dimer was 0.89 mg/L, which was statistically different (*P* = 0.000). D-dimer is a fibrin degradation product, which is usually elevated in the context of DVT. D-dimer shows high sensitivity but low specificity in diagnosing VTE. It is often also elevated in inflammation, malignancy, and other systemic diseases and is a non-specific indicator (43). It has been shown that D-dimer levels are strongly associated with the development of VTE within specific patient groups. A study on risk factors for VTE in lung cancer patients found that a pre-chemotherapy D-dimer concentration of ≥1.44 mg/mL was significantly associated with the occurrence of VTE events in patients with non-small cell lung cancer (44). In another study evaluating the risk of VTE in COVID-19 patients, researchers developed a multivariate predictive model that incorporated D-dimer. This model confirmed the predictive value of D-dimer for VTE events in patients with infectious diseases (45). Our study found that plasma D-dimer levels in CHD patients who experienced VTE events were significantly higher than in those who did not develop VTE. This result suggested that plasma D-dimer levels in CHD patients could predict the occurrence of VTE events.

In the present study, the median age of the patients in the VTE group was significantly higher than that in the non-VTE group (81 vs. 75 years), with a statistically significant difference (Table 2). CHD is a common disease in the elderly population. Some studies have also shown that the incidence of VTE increases with age (21). We had similar findings in the present study. Increasing age increases the risk of VTE in CHD patients. Older patients with CHD are associated with more VTE-related risk factors, and plasma concentrations of coagulation factors increase with age (5). This change in risk factors and coagulation factors explains why age is an independent risk factor for VTE in CHD inpatients.

Another finding of this study was that the level of NEUT was significantly higher in VTE patients than in non-VTE patients (5.59 × 10^9^/L vs. 4.77 × 10^9^/L), with a statistically significant difference (Table 2). Multiple studies have investigated the correlation between NEUT and VTE events (46–48). In a study of sepsis patients, researchers found that neutrophil extracellular traps (NETs) can promote hypercoagulability in these patients (49). Additional research has also identified plasma citrullinated histone H3 (CitH3) as a biomarker for NET formation (50). Patients with symptomatic VTE exhibit elevated plasma CitH3 levels and accelerated thrombin kinetics (51). These findings suggest that NETs may serve as an intermediary link between NEUT and VTE development.In the present study, we found that NEUT levels could be a risk factor for the development of VTE in hospitalized patients with CHD. It is noteworthy that clinicians usually focus on the correlation between age, D-dimer, and VTE while ignoring the correlation between high NEUT levels and VTE events, especially for CHD inpatients. Clinicians should pay attention to NEUT levels in CHD hospitalized patients to prevent VTE in advance.

This study represented the first investigation into predictive models for the occurrence of VTE in hospitalized patients with CHD. Given the convenience and low cost of tests for indicators such as D-dimer and NEUT, which are readily accessible in primary healthcare settings (52, 53), machine learning models based on these variables demonstrate strong potential for widespread application. Future research will explore intervention thresholds for VTE in specific study populations to enhance the model's applicability (54). Of course, this study also had several limitations. Firstly, we did not analyze the data of patients with PE separately, mainly because the number of patients with a diagnosis of PE was small in this study. We found that all patients with PE had concomitant DVT, and therefore, we did not conduct a separate study on PE. Second, the optimal models obtained in this study were inconsistent between the internal and external validation sets. Although the performance of LR + LASSO in the external validation set was better than that of XGBoost, the number of samples in the external validation set in this study was significantly lower than in the internal validation set. Therefore, we believe that XGBoost may be more stable than the LR + LASSO method. Due to the small sample size of the external validation set, this study might be subject to selection bias, which could affect the model's generalizability. In the future, we will expand the sample size to improve model stability and increase the number of variables to make the machine learning model comparable to existing scoring systems (such as Padua and Caprini).

In summary, machine learning models (XGBoost and LR + LASSO) perform well in predicting VTE in hospitalized CHD patients using easily obtainable characteristics at admission. Machine learning algorithms can help clinicians screen for VTE and dynamically monitor changes in the condition of VTE-prone populations.

## Data Availability

The data analyzed in this study is subject to the following licenses/restrictions: The data for this study were obtained from the Benxi Central Hospital in Liaoning Province and the Benxi Clinical Biospecimen Bank. The dataset is a non-public use dataset due to the patient information involved. In this study, patient records and information were anonymized and de-identified prior to data analysis. Readers who wish to review and exchange data analyzed in this study may contact the corresponding author of this article. Requests to access these datasets should be directed to Hui He, Email: lnbxwxf@yeah.net.
